# A Rare Cause of Colonic Obstruction: Inflammatory Fibroid Polyp

**DOI:** 10.7759/cureus.23868

**Published:** 2022-04-06

**Authors:** Sevinc Dagistanli, Nermin Gunduz, Osman Sibic, Suleyman Sonmez

**Affiliations:** 1 General Surgery, Health Sciences University, Kanuni Sultan Suleyman Training and Research Hospital, Istanbul, TUR; 2 Pathology, Health Sciences University, Kanuni Sultan Suleyman Training and Research Hospital, Istanbul, TUR; 3 Interventional Radiology, Health Sciences University, Kanuni Sultan Suleyman Training and Research Hospital, Istanbul, TUR

**Keywords:** hemicolectomy, polyp, fibroid, inflammatory, invagination

## Abstract

Only 5% of all cases of intussusceptions occur in adults. Although it is known to occur frequently due to inflammatory bowel disease, postoperative adhesions, or neoplastic masses, inflammatory fibroid polyps (IFP), which are rare lesions of the gastrointestinal tract, may present this clinical picture. In rare cases of intussusception due to IFP, clinical suspicion should be kept in the foreground and mind in the differential diagnosis. In this article, the purpose was to present the clinical, radiological, and pathological findings and the treatment of obstruction caused by this rare lesion.

## Introduction

Intussusception is the condition that appears when the proximal bowel loop is invaginated into the distal intestinal loop lumen. Its prevalence is only 5% in all invagination cases in adults. There is often an underlying organic lesion [1]. It often occurs as a result of inflammatory bowel disease, postoperative adhesion, or benign/malignant neoplasms [2, 3]. Inflammatory fibroid polyps (IFPs) are rare lesions of the gastrointestinal tract, and cause invagination and related obstruction, and are usually diagnosed during surgery. IFPs must be kept in mind in the differential diagnosis of invagination [4].

## Case presentation

**Figure 1:**
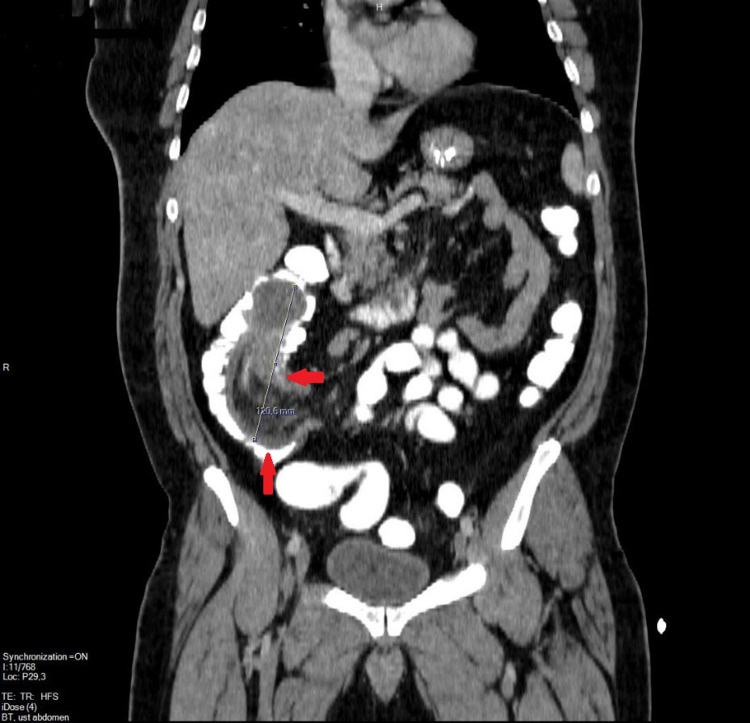
The invaginated intestinal segment at the cecum-ascending colon level in the lower-right quadrant of the abdomen (Red Arrow: Ileocolic intussusception)

A 40-year-old male patient presented to the Emergency Department in March 2021 with complaints of common abdominal pain and accompanying nausea, vomiting and no defecation that had lasted for four days. He had no known history of additional disease. He only had a history of surgery because of lumbar disc herniation. Signs of abdominal distension were detected in physical examination. Defense and rebound were not detected. The laboratory examination results were as follows: C-reactive protein was 57.84 mg/L (normal: <5 mg/L), leukocytes were 12.84 (normal: 3.8-10 × 10^9^/L) in the blood cell count, and other blood parameters were normal. There were one/two air-fluid levels in the abdominal X-ray, the non-contrast abdominal tomography performed for further examination revealed an appearance that was compatible with ileocolic invagination of the hypodense appearance of the intestinal loop in the peripheral area and with mesenteric fatty tissue in the center (Figure 1).

**Figure 2:**
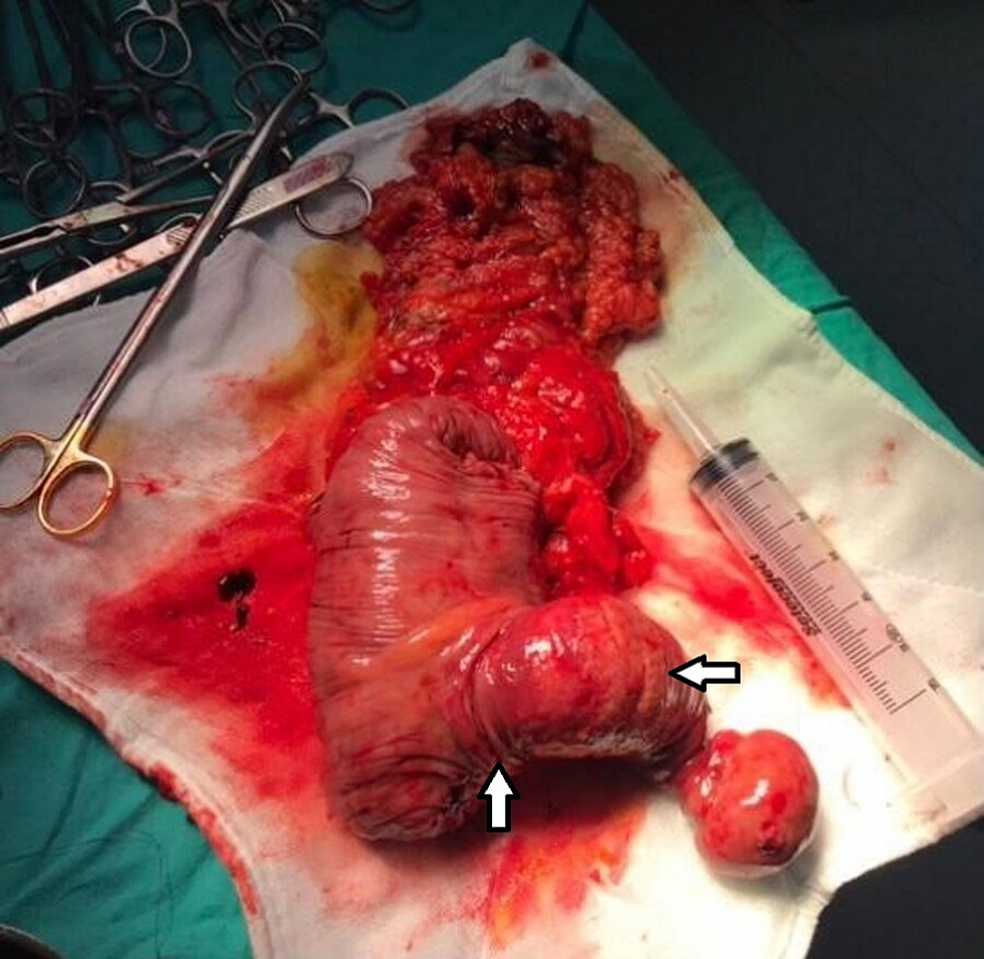
Postoperative image of the invaginated bowel segment (White Arrow: Ileocolic intussusception on postoperative specimen)

A filling defect in the lumen was noted in the proximal soft tissue density. A minimal dilatation with a 3-cm diameter was detected in the small intestinal loops. When findings consistent with invagination and ileus were detected, urgent surgery was planned for the patient. It was observed during the exploration that the segment located 10 cm distal to the ileocecal valve was invaginated into the ascending colon along with the ileocecal valve. Dilatation had developed because of obstruction proximal to the invagination. When a solid mass was detected in the ascending colon, the patient underwent right hemicolectomy and end-to-side anastomosis (Figure 2).

**Figure 3:**
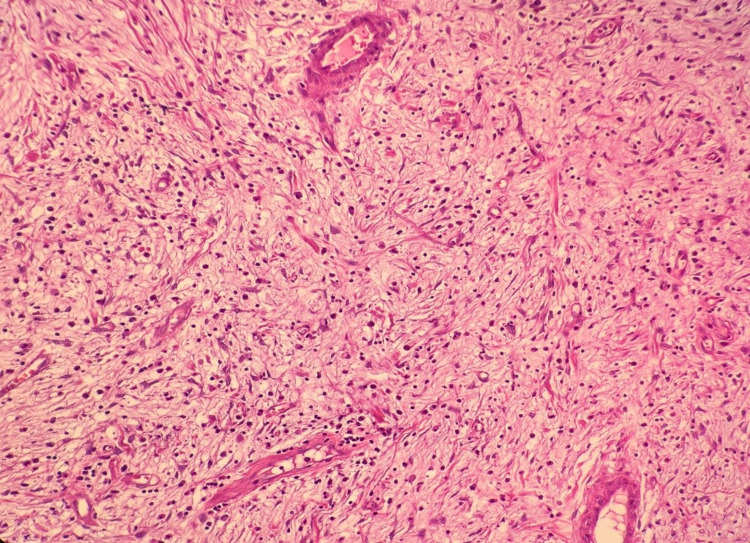
Microscopic image of inflammatory fibroid polyp (H&E, x100)

**Figure 4:**
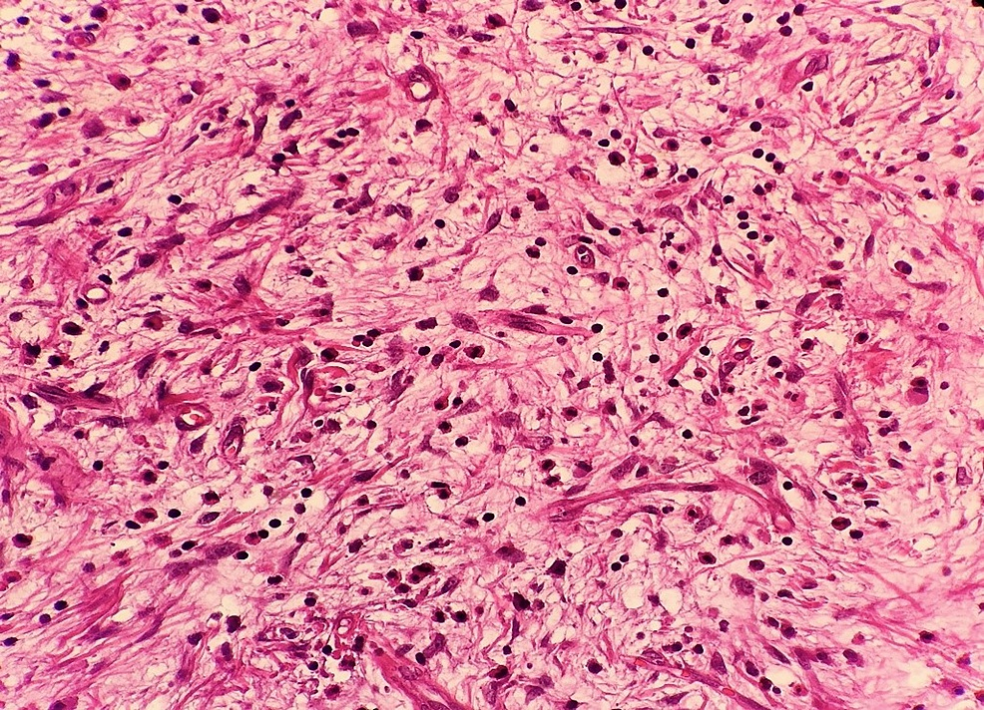
Microscopic image of inflammatory fibroid polyp (H&E, x400)

**Figure 5:**
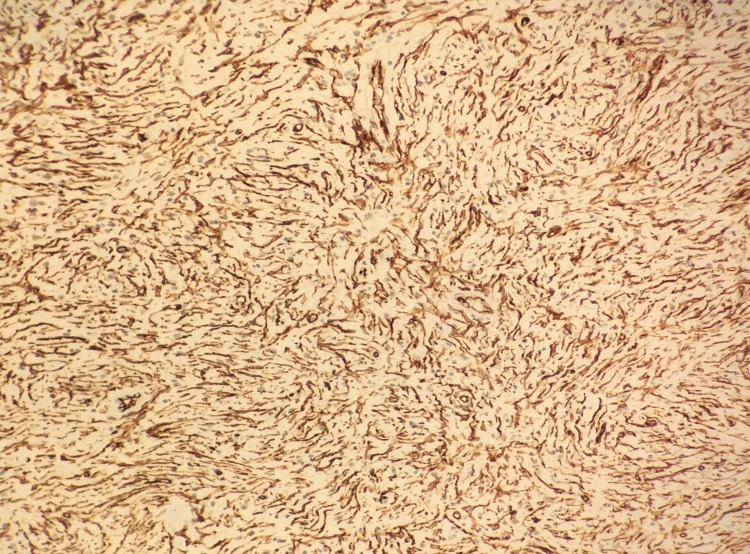
Positive staining with CD34 (x100)

The patient was discharged on the 7th postoperative day with full recovery. The distal 50 cm ileal segment was examined in the colonoscopic examination in the postoperative 4th month, and no pathological finding was found. The specimen that was extracted macroscopically consisted of a 9-cm-long small intestine and 23-cm-long colon resection material. When the material was opened, an 11x5x4 cm polypoid lesion was found, which originated from the ileum and which invaginated the colon lumen with the ileocecal valve. It was observed on the section surface that this area consisted of a 4.5-cm diameter yellow-white colored nodular solid lesion distally and there was an invaginating small intestine segment. It was observed in the microscopic examination of the samples that were taken from the lesion that the surface was covered with focal ulcerated small intestinal mucosa. In the submucosal area, inflammatory cells rich in eosinophils, vessels, spindle, and stroma that consisted of stellate cells were observed. Atypia, mitosis, and necrosis were not observed. In the immunohistochemical examination, positive immunoreactivity was detected with CD34, and negative immunoreactivity with CD117, S-100, Desmin, Dog-1 in spindle cells (Figures 3-5). A diagnosis of inflammatory fibroid polyp was made based on the histomorphological and immunohistochemical findings. The polyp also had a stem.

## Discussion

One of the rare causes of intestinal obstruction is invagination. IFPs are one of the rarest causes of invagination in adults. IFPs were first reported in 1949 as “gastric submucosal granuloma with eosinophilic infiltration” [5]. Although many different definitions were used for the lesion over the years, the definition of IFP began to be used in 1953 [2]. Invaginations are evaluated in three categories as ileoileal, ileocolic, and colo-colic. Invaginations are more common in adults than small bowel colon invaginations [2]. Invagination is a condition that develops against anatomical obstacles in the direction of intestinal peristalsis [5]. Ileocecal (colic) invagination was detected in our patient.

IFPs can be observed in many parts of the gastrointestinal tract. The most common area is the antrum, which is followed by the small intestines. Colorectal IFPs are seen between 4 and 7% [2]. Ileum-localized IFP was detected in our case [6]. The etiology of IFPs is unclear. They can be caused by traumas, allergies, neoplastic processes, and bacterial, physical, chemical, or inflammatory stimuli [2]. Although there are explanations of the trauma effect of the food contents in the most common gastric lesions, it was not considered as a suitable mechanism for explaining the cause of IFP in our case [2].

IFP is an asymptomatic lesion, and polyps in the small intestine or colon give symptoms with signs of invagination or obstruction. Nausea, vomiting, diarrhea, bloody stools, and tenesmus may also accompany these clinical conditions [2]. The age at which IFPs occur in patients is reported as 60-70 years in the literature. Lesions are usually defined in sizes that range between 2 and 5 cm, and cases of larger diameter were also reported rarely. They can be with or without stems [7]. In our case, the polyp was 4.5 cm in diameter and had a stem. However, it was seen macroscopically as a polypoid mass of 11x5x4 cm, which caused obstruction in the colon lumen as it showed invagination with the small intestine segment from which it originated.

The first tool used in the diagnosis of invagination was the standing direct abdominal radiography; however, it is generally insufficient to make a specific diagnosis. Double-contrast imaging methods are more useful in diagnosis. Colonoscopy can also be used for diagnosis in colonic IFPs, but in the literature, it is reported that invagination develops over IFP after colonoscopy [1, 8]. The malignant potential of the lesion that was removed after the curative surgery was not reported. Only one recurrence case was reported in the literature.

## Conclusions

Obstruction due to inflammatory fibroid polyp is a rare phenomenon. The underlying cause may be benign, malignant, or iatrogenic. High clinical suspicion is required to make this rare diagnosis in patients presenting with classical ileus. It is important to reveal this lesion, which has no potential for malignancy, with appropriate imaging and diagnostic techniques, to perform appropriate treatment methods, and to examine the postoperative specimen for possible additional diagnoses with appropriate pathological methods.
